# Prosodic Transfer in English Literacy Skills among Chinese Elementary-Age Students: Controlling for Non-Verbal Intelligence

**DOI:** 10.3390/jintelligence10040114

**Published:** 2022-11-25

**Authors:** Jiexin Lin, Haomin Zhang, Xiaoyu Lin

**Affiliations:** 1 The Psycholinguistics Lab, School of Foreign Languages, East China Normal University, Shanghai 200050, China; 2 The Foreign Language Teaching and Research Center, School of Foreign Languages, East China Normal University, Shanghai 200050, China; 3 School of Cultural Creativity and Management, Communication University of Zhejiang, Hangzhou 310019, China

**Keywords:** lexical tone, stress, prosodic transfer hypothesis, English oral vocabulary, English word reading, English word spelling

## Abstract

Building upon the prosodic transfer hypothesis, the current study aims to examine the intermediary effect of English stress on the relation between Chinese lexical tone awareness and English word-level literacy (reading and spelling) as well as the moderating effect of English oral vocabulary proficiency on the cross-linguistic association. Grade 4 Chinese learners of English (*N* = 224) participated in this study and were assessed for their tone and stress sensitivity, English oral vocabulary, English word reading, and English word spelling. Mediated multivariate analyses with moderation were used to explore: (1) whether the influence of lexical tone perception on L2 word reading and spelling was mediated by English stress as posited in the prosodic transfer hypothesis; (2) whether the effects of tone on English word reading and spelling performance varied as a function of oral vocabulary levels. The findings revealed a direct positive relationship between Chinese tone and English word reading and spelling, and the relationship was mediated by English stress awareness. Furthermore, the direct pathway from tone to English word-level literacy skills were moderated by oral vocabulary and the relationship between tone and English word-level skills became stronger as oral vocabulary levels increased; however, such strength reached a plateau among children without adequate oral vocabulary skills. These findings suggest the necessity to incorporate word spelling as an outcome in the cross-suprasegmental phonological transfer models of early literacy development. Additionally, the current study endorses the complexity of cross-language prosodic transfer. It points to a precise threshold for sufficient L2 oral vocabulary skills to enable tone transfer in English word-level literacy attainment.

## 1. Introduction

Biliteracy development is inherently more complex than monolingual acquisition (Koda et al. 2014). Understanding the multi-layered complexity inherent in biliteracy development is becoming increasingly important. Since the 90s, there has been a dramatic proliferation of research concerned with cross-language transfer to explore how reading sub-skills are assimilated in learning to read in an additional language. Substantial evidence has supported the cross-linguistic transfer of phonological awareness in either L1 or L2 reading skills among learners of diverse language pairings (e.g., Gottardo et al. 2001; Gottardo et al. 2006). Phonological awareness has been regarded as a language-general construct (Shany et al. 2010). Nonetheless, there is no consensus as to the patterns of transfer. Indeed, transfer is a complex process that encapsulates analyses of units varying in complexity and that is contingent upon multiple factors (Chung et al. 2019). Clearly, relative levels of proficiency in the L1 and L2 are one of the determining factors in cross-language transfer. However, a dearth of empirical research has specified the exact role of language proficiency in the transfer process. Data from correlational studies between phonological transfer has reported vocabulary as a potential moderator (Atwill et al. 2010). To further specify the extent to which the potential moderator may impact the phonological transfer, we see a need to conduct a moderator analysis by including an interaction term between the predictor and moderator variable. Additionally, given that language distance between L1 and L2 is also a contributing factor to cross-language transfer (Chung et al. 2019), exploring transfer in the typologically distant language pairings such as English (alphabetic script) and Chinese (logographic script) may provide a more interesting lens to better seize different patterns of transfer across scripts.

With respect to phonological transfer, many empirical data have implicated the pivotal role of segmental phonological awareness in reading. It is legitimate because of the widespread acknowledgment of segmental phonological awareness in early reading skills and the deficits in segmental phonological processing are causally linked to poor word-identification skills (Bus and van Ijzendoorn 1999; Stanovich and Siegel 1994). Recently, research has begun to investigate the potential impact of suprasegmental sensitivity (prosodic sensitivity) and confirmed a substantial support of the cross-language association between suprasegmental phonological awareness and dual-language reading development (e.g., Tong et al. 2016). Prosodic awareness refers to the ability to identify and manipulate acoustic properties across speech segments including stress, intonation, and timing (Holliman et al. 2017). Indeed, Protopapas et al. (2006) have claimed that “reading models must be extended to account for multisyllabic word reading including, in particular, stress assignment” (p. 418). Prosodic sensitivity contributes to reading by facilitating parsing spoken language into syntactic information for comprehension (Gleitman et al. 1989; Kuhn and Stahl 2003), during which the memory load is reduced and thereby efficient information retention is achieved at ease (Koriat et al. 2002; Speer et al. 1993). The shared overlapping neural networks employed in prosody perception allow for prosodic sensitivity to transfer across scripts (Patel 2011, 2014). However, as mentioned previously, cross-language transfer is an inherently complex process influenced by multiple factors. Vocabulary has been considered a potential moderator that impacts on cross-language phonological awareness transfer (Atwill et al. 2010). Additionally, the prosodic sensitivity that is known to influence L2 reading may not contribute equally for children with different levels of oral vocabulary, which invites scrutiny to identify how the influence of prosodic sensitivity associated with L2 reading-related skills may vary depending on the level of vocabulary knowledge. However, relatively few studies have addressed the possible moderating effect of vocabulary on the prosodic transfer process. In the context of Chinese–English bilinguals, research interest has increased to address phonological awareness transfer at the suprasegmental level in L2 reading-related skills, such as vocabulary (e.g., Choi et al. 2019b; Tong et al. 2016) and reading comprehension (e.g., Choi et al. 2016). However, scant research has probed into word-spelling outcomes. Additionally, much previous work has focused on Cantonese–English bilingual children, while ignoring Mandarin–English bilingual children. These two participating groups differ in terms of linguistic backgrounds and suprasegmental features in their source language, which are considered critical factors to impact the patterns of cross-language transfer (Chung et al. 2019). To be specific, Cantonese–English bilingual children simultaneously learn Chinese and English at a very young age, while Mandarin–English bilingual children start learning English in Grade 1 or as late as Grade 3; Cantonese exhibits a much more complex tonal system with six lexical tonal tones than Mandarin with only four lexical tones. Therefore, it seems imperative to evaluate how the prosodic transfer works among Mandarin learners of English in a tonal language with a less complex tone system.

Building on this background, the current study aims to investigate whether prosodic sensitivity can have a role in L2 word-level literacy (reading and spelling) among Mandarin-speaking children, and how vocabulary influences the pathway from prosody to word-level literacy. Against the backdrop of an Anglocentric focus, we move beyond to identify the cross-language transfer pattern among typologically dissimilar language pairings. The demystification of the pathways underlying Chinese (logographic) prosodic transfer in English reading attainment may help to enrich cross-suprasegmental phonological transfer models of early literacy development. Through the investigation into the moderating effect of vocabulary on prosodic transfer, this study may add to the current understanding of the complexity in cross-language transfer and identify the exact role of vocabulary proficiency in the transfer process. Additionally, this study can help make inroads into educational practice. This study is interested in specifying the extent to which vocabulary may impact cross-suprasegmental phonological awareness transfer in L2 word-level literacy. It may inform educators to adopt different instructional programs to meet the specific needs of distinct types of reading profiles.

### 1.1. Lexical Tone Awareness in English Word Reading and Spelling

In the context of English and Chinese bilingual learners, literature has begun to address the importance of lexical tone in L2 (English) reading attainment (e.g., Choi et al. 2016; Zhang and Koda 2021; Zhang et al. 2022; Tong et al. 2016). However, variations exist in the pathways driving prosodic transfer, and the proposed candidate-mediating mechanisms include vocabulary growth, stress, segmental phonological awareness, and morphological awareness (Holliman et al. 2017). The prosodic transfer hypothesis is one of the conceptual prosody transfer models that underpin the mediating role of English stress in the relationship between lexical tone and English word reading (Tong et al. 2016; Wang et al. 2009). To date, whether such prosodic transfer may also have a role in word spelling is less explored. Wood et al. (2009) first modeled the relationship between prosodic sensitivity and early L1 English word reading and spelling. They attested to the intermediary effect of vocabulary, segmental phonological awareness, and morphological awareness on literacy skills. Nonetheless, whether prosodic sensitivity can facilitate L2 reading and spelling is under-explored. Reading and spelling are interconnected constructs in an early development stage (Fitzgerald and Shanahan 2000). The close connection between word reading and spelling has also been observed in previous studies (Lin et al. 2017). Spelling, a highly analytical process, encompasses phoneme decoding, letter-sound knowledge recalling, and attention to the shapes, names, and sequences of the individual letters (Shahar-Yames and Share 2008). It provides an interface for connections between phonological, orthographic, and semantic knowledge (Lin et al. 2017). Additionally, word reading requires mapping sounds (phonology) onto letters (orthography). Furthermore, learning to spell provides learners with a concrete opportunity to practice, thereby consolidating the connection between orthography and phonology (Shahar-Yames and Share 2008). Lastly, semantic knowledge of the words has been observed to further consolidate the phonological–orthographic link and facilitate spelling accuracy (e.g., Hilte and Reitsma 2011; Ouellette 2010). Lexical tone and stress can provide prosodic cues to distinguish meanings (Deng et al. 2019; Tong et al. 2015). To be specific, lexical tone, a prominent feature in Chinese, refers to the different pitch patterns. There are four lexical tones in Chinese, and the variations of tonal intonations can distinguish different meanings of an identical monosyllable. For instance, the monosyllable /xi/ representsfour different meaningswhen pronounced with different patterns: /xi^1^/ 西 (clothing), /xi^2^/ 媳(daughter-in-law), /xi^3^/ 喜(adore), and /xi^4^/ 细 (slim) (the superscript numbers indicates tone patterns). Lexical Stress refers to the variations of pitch, duration, intensity, and vowel quality (Fry 1958). These prosodic cues can help signal the prominence of a syllable. A strong–weak syllable guides the segmentation of the speech stream (Cutler and Norris 1988; Vroomen et al. 1998) and triggers semantic activation. For instance, the meanings of English words can be distinguished on the basis ofsyllabic prominence, such as “DEsert” and “deSERT”. In that way, prosodic sensitivity may also enhance spelling by reinforcing semantic information. Taken together, it stands to reason to assume that prosodic sensitivity may also have a consequence in word spelling.

**Figure 1:**
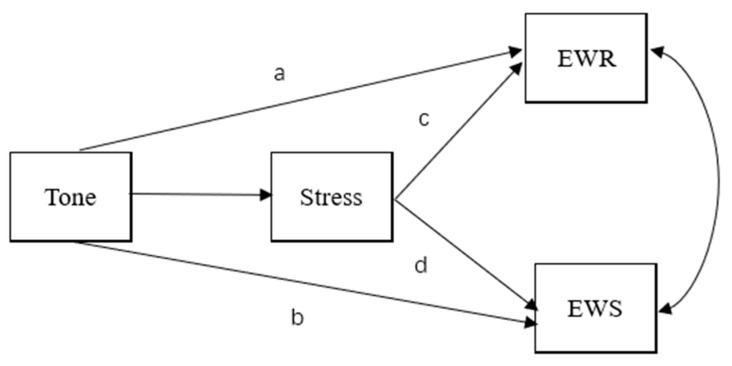
Hypothesized model for the relation between tone, stress, English word reading and spelling. Tone = Chinese tone sensitivity; Stress = English lexical stress sensitivity; Voc = English oral vocabulary; EWR = English word reading; EWS = English word spelling. Direct ways are: (**a**) Tone→EWR; (**b**) Tone→EWS. Indirect ways are: (**c**) Tone→Stress→EWR; (**d**) Tone→Stress→EWS.

Building on previous explorations, we proposed a conceptual model to delineate the influence of cross-linguistic prosodic sensitivity on L2 word reading and word spelling. We hypothesized that L1 lexical tone awareness contributes to L2 word reading and spelling both directly and indirectly through English stress (depicted in Figure 1). There are two direct tone transfer pathways, and two possible indirect prosodic transfer pathways. The direct tone transfer pathways from lexical tone to L2 word reading and spelling align with the transfer facilitation model (Koda 2008), which recognizes the well-established L1 competencies in shaping the development of reading skills in another language (a. Tone→English word reading; b. Tone→English word spelling). The hypothesis was also supported by empirical data which indicated the unique contribution of prosodic sensitivity to L2 word reading after incorporating the precursors of vocabulary, segmental phonological awareness, and morphological awareness (e.g., Holliman et al. 2017). However, as opposed to word reading, prosodic sensitivity was not observed to play a unique role in word spelling on top of these well-established precursors. Therefore, in the bilingual context, prosody may transfer to L2 word-level literacy skills in an indirect manner. Together with the prosodic transfer hypothesis (Tong et al. 2016; Wang et al. 2009), we hypothesize that English stress links lexical tone awareness with L2 word-level abilities (c. Tone→Stress→English word reading; d. Tone→Stress→English word spelling). The close association between tone and stress is grounded in the shared acoustic properties and linguistic functions. The common perceptual mechanism of precise F0 tracking is needed in prosodic perception (Choi et al. 2017, 2019a). Additionally, it is widely accepted that tone and stress are two principal ways by which meanings are distinguished on the basis of the prosodic cues (Cutler and Chen 1997, p. 165). The interconnection of Chinese and English prosodic perception makes it compelling to examine the role of English stress in the tone transfer pathway. Evidence to support the mediating role of stress in the relationship between tone and L2 reading also stems from recent studies (e.g., Choi et al. 2019b).

### 1.2. The Impact of Oral Vocabulary on Prosody-Literacy Relationship

As discussed, the role of lexical tone in L2 English literacy has been broadly assessed, yet variations exist as to the manner by which tone affects L2 reading development. Given that “sufficient language proficiency” and “automaticity” has been much emphasized for the transfer to occur (Cummins 1981, 2012; Koda 2008), one plausible underlying reason driving the different pathways is language proficiency in reader profiles. Oral vocabulary has been considered to be a typical characteristic of reading proficiency (Joshi 2005). An array of studies has implicated a pivotal role of receptive oral vocabulary skills in forming the initial basis of reading skills among L1 and L2 learners (e.g., Zhang 2016; Zhang and Koda 2021), and the variability of oral language proficiency may engender different patterns of reading abilities (Zhang and Koda 2021). Intriguingly, an additional body of research has pointed out that oral vocabulary may impact on the degree to which reading-related variables influence reading attainment (e.g., Lee and Chen 2019). For instance, Riedel (2007) found that fluency significantly predicted reading comprehension only among students with adequate vocabulary skills. Lee and Chen (2019) directly examined the interaction of reading fluency and vocabulary and indicated a unique within-language contribution of the interaction in reading comprehension among Grade 3 English–French bilingual students. Following this line of inquiry, the nuanced relationship between reading-related predictors (i.e., prosody) and reading may vary as a function of oral vocabulary. Although prosodic sensitivity has been included as an aspect of oral reading fluency (Schwanenflugel and Benjamin 2017), to our knowledge, little research has directly examined the role of prosody in reading attainment by including the moderation of oral vocabulary. Furthermore, most studies on the interaction of multiple reading-related factors have been conducted among monolingual students, yet much less is understood with regard to language-diverse students. Indeed, Lee and Chen (2019) explored English–French bilingual students, however, their study did not focus on the interaction of fluency and vocabulary in predicting cross-language reading achievement. In addition, Atwill et al. (2010) reported in their correlation analysis that vocabulary was a potential moderator on segmental phonological awareness (measured in phonemic awareness) transfer on Spanish–English bilingual children. They focused on the transfer process between phonological awareness and did not explore its moderating effect on phonological transfer in L2 reading achievement. Therefore, it seems critical to explore how the oral vocabulary profile of each child may impact on the link between suprasegmental (prosodic) information and dual-language reading ability, and to quantify the specific role of vocabulary in moderating the transfer pattern using moderation analysis. This question is of particular importance given that vocabulary may exert a strong influence on reading proficiency among language-diverse readers (Lervåg and Aukrust 2010). Specifically, L2 learners typically fall behind their L1 peers in vocabulary knowledge and consequently lag on L2 reading comprehension (e.g., Farnia and Geva 2011). More importantly, growth in vocabulary supports gains in phonological awareness through the lexical restructuring process (Metsala and Ehri 1998). To be specific, as vocabulary accumulates, children will be more engaged in distinguishing existing word repertoire stored in memory from newly encountered words for successful recognition. In this way, children will gain more fully specified and/or segmental lexical representations, and such phonological manipulation or restructuring of lexicon facilitates phonological awareness (Metsala and Ehri 1998). Moreover, the interaction of multiple factors appears to be a cause facilitating the transfer of a certain construct (Chung et al. 2019). Building on this background, the second aim of the current study is to explore whether the influence of tone sensitivity on L2 word-level literacy varies based on vocabulary levels. Theoretically, it scrutinizes the complexity of cross-language transfer of phonological awareness and helps identify a linguistic threshold (indexed by oral vocabulary) to enable the transfer of tone sensitivity in L2 English reading development.

In summary, a growing body of literature has demonstrated that lexical tone is positively linked to early L2 (English) literacy development; yet, the precise nature of this cross-linguistic link remains controversial. It has been speculated in the prosodic transfer hypothesis (Tong et al. 2016; Wang et al. 2009) and recent studies (e.g., Choi et al. 2019b; Tong et al. 2016) that tone indirectly contributes to L2 word reading via English stress. However, how prosodic sensitivity transfers in L2 word spelling is less understood. Moreover, given that cross-language transfer is interactive in nature, the transfer of a certain construct seems to be induced by the interaction of multiple units of analyses (Chung et al. 2019). Together with the evident role of vocabulary in L2 literacy, it seems compelling to examine how oral vocabulary skills moderate the relation between prosodic perception and L2 word-level literacy skills (reading and spelling).

### 1.3. Objectives of the Present Study

The study uses mediation analysis first to disentangle the tone transfer in English word reading and spelling ability to expand our understanding of the prosodic transfer hypothesis from L2 word reading to word spelling skills. It is also important to determine if the cross-linguistic influence of prosodic sensitivity on L2 word reading and spelling is moderated by the level of oral vocabulary. Evidence of cross-language transfer between phonology has indicated that whether the language spoken at home matches the language of instruction is another potential moderator (e.g., Kim et al. 2016; Uwezo 2011). So, we evaluated L2 oral vocabulary instead of L1 oral vocabulary as the potential moderator because English is not commonly spoken outside of classrooms for the participating Chinese children. Thus, the interference of the language spoken at home with oral vocabulary can be lessened to a greater degree, thereby promoting the efficacy of the moderating effect in itself. Additionally, the threshold for cross-language transfer has been conventionally conceptualized in the language proficiency in the source language (Cummins 1981, 2012; Koda 2008). Children with stronger L1 proficiency show a tendency for L1-to-L2 transfer. Nonetheless, cross-language transfer is inherently complex and biliteracy competence is multi-faceted (Koda et al. 2014). It is also of great significance to explore whether language proficiency in L2 may also influence the cross-language pattern. The unspecified threshold in models of cross-language transfer also motivates our investigation of themoderating effect of vocabulary.

To summarize, there are two research questions addressed in the current study.

1. Does lexical tone awareness have direct or indirect effects on English word reading and spelling through lexical stress sensitivity, after controlling for the effect of non-verbal intelligence?

2. Does L2 oral vocabulary moderate the prosodic transfer in English word reading and spelling, after controlling for the effect of non-verbal intelligence?

## 2. Materials and Methods

### 2.1. Participants

The participating children were 224 native Mandarin Grade 4 students (100 girls, 124 boys; mean age = 10.148 years, SD = .36 years) from a public school in the Zhejiang Province, who started to receive formal Chinese instruction in first grade and began learning English in third grade. None of the participants had been identified as having developmental language disorders or brain damage. This age group was selected because their perceptual systems were sufficiently developed to perform the tasks used in the current study and because their proficiency level was shown to be appropriate for the English reading and spelling tasks, especially for the pseudo-word items after having received formal English literacy instruction for more than one year. Their English curricula adhered to the guidelines of the criteria put forth by the Ministry of Education of the People’s Republic of China (2021). By the end of the first semester of Grade 4, students should be able to identify or recognize the pictures or objects based on the words they hear; imitate English from the recording, read words, and write letters and words correctly. They are supposed to recognize 400–700 English words by the end of Grade 4. Additionally, the participating age group was in the transitional stage of literacy acquisition between “learning to read” and “reading to learn” (Chall 1996), during which the importance of vocabulary was particularly evident (Chall 1983).

### 2.2. Instruments

#### 2.2.1. Non-Verbal Intelligence

Non-verbal reasoning ability was assessed using Raven’s Standard Progressive Matrices (Zhang and Wang 1985). This test consisted of 5 sets of 12 items with each set arranged in graded difficulty. Children had to select the best-fitting item for the matrix from a choice of 6 pictures. 1 point was awarded for each correct response. The maximum score was 60 points. The Cronbach’s alpha of this measure was .801.

#### 2.2.2. Chinese Tone Awareness

Chinese tone awareness was assessed using the tone discrimination task. This format was used considering that no ceiling effect was found in the discrimination task, and zero inclusion of speech production (Burnham et al. 2011). It comprised 2 practice items and 10 test items, with five tonal contrasts (i.e., T1-T2, T1-T3, T1-T4, T2-T3, T3-T4). In each test item, children were presented with three tonal monosyllables. Two of them carried the same tone, and one a minimally different tone. Each tone contrast was repeated two times. All test items were auditorily presented to children, such as A. /gao^1^/ (*tall*), B. /ba^4^/(*father*), C. /dai^1^/ (*stay*) (the superscript numbers indicated tone patterns) Children then needed to distinguish different lexical tone contrasts by choosing which of the tonal syllables was pronounced in a different way from the other two. In this case, the tone intonation for “B. ba^4^” was “falling down”, different from the other two, so B should be selected. The three words for each item were high-frequency words or commonly used in daily life. One point was given for each correct answer, for a maximum of ten. The Cronbach’s alpha of this measure was .721.

#### 2.2.3. English Stress Awareness

We assessed English stress awareness using the stress discerning task. It comprised 2 practice items and 10 test items. The test items were trisyllabic or quadrisyllabic words, 5 of which were assigned with reversed syllabic stresses, and the remaining 5 with unreversed syllabic stresses. We presented them auditorily, and the stress contrasts were repeated two times. Children were told that the words that they heard were not always spoken correctly. They needed to circle the stressed syllable that corresponded with what they heard on the answer sheet. Note that all multisyllabic words without reversed syllabic stress were unknown to the children, to control for the possible familiarity effect of vocabulary knowledge. Items with reversed syllabic stress involved a change in the lexical stress such that the stress fell on the incorrect syllable (e.g., toMAto becomes tomaTO). One mark was given for each correct answer, for a maximum of ten. The Cronbach’s alpha of this measure was .700.

#### 2.2.4. English oral Vocabulary

The English oral vocabulary task was adopted from the Peabody Picture Vocabulary Test (PPVT; Dunn and Dunn 1997). There was a total of 20 sets of test plates. Each set was composed of four pictures. They were black-and-white illustrations of common objects, food, animals, actions, and so forth. In this task, children needed to select the picture corresponding to the semantic meaning of the prerecorded word by the experimenter. Testing items were selected out of the first thirty items in the PPVT (e.g., teacher, car). One point was assigned to each correct answer, for a maximum of twenty. The Cronbach’s alpha of this measure was .720.

#### 2.2.5. English Word Reading

Real word and pseudo-word reading subtasks were used to evaluate children’s ability to read aloud printed single words. Children were required to correctly read each word as quickly as possible from a printed list of 20 real words and 16 pseudo words arranged in ascending difficulty. The real words were selected from their English curriculum, and the pseudo words were adapted from Word Attack Test (Woodcock 1998) and adjusted to be appropriate for Grade 4 students. The pseudo-word subtest was preceded by two practice trials to ensure children’s complete understanding of the task instructions and the application of grapheme–phoneme correspondence rules. Testing was halted following 15 consecutive failed items. Children were given 1 point for each correctly pronounced word, for a maximum of 36. The Cronbach’s alpha of this measure was .755.

#### 2.2.6. English Word Spelling

This was assessed with a spelling test consisting of 10 real words and 10 pseudo words. Participants were required to correctly spell the words they heard on a given piece of paper. The real words consisted of 8 one-syllable and 2 two-syllable words, all of which were selected from their English curriculum and considered to be orally familiar to Grade 4 students. The pseudo words were designed based on the sight words (e.g., the target “tog” was selected from the clue word “dog”). Children received 1 point for each correct spelling of the whole word, and .5 points for the partially correct spelling. The .5 scale was used considering that the participants could not distinguish some similarly pronounced sound pairs, such as between “/æ/” and “/e/”. The Cronbach’s alpha was of this measure .863.

### 2.3. Procedures

The study was approved by the ethics committee in the researchers’ institution. All measures were given to children after informed consent was obtained from schools and parents. The English word reading test was administered individually to the participants for approximately 10–20 min. The remaining tests were administered collectively within schools during the children’s normal class time in two time slots within a 2-week range. The Chinese tone sensitivity tasks and English tasks (except for English word reading) were completed on two separate days. The testing order was randomized to rule out the learning effect from the previous tasks. Half of the participants were tested first with the Chinese tone awareness task, whereas the other half were tested first with English tasks. Testing took place in the middle of the first semester, that is, November through December.

## 3. Results

### 3.1. Preliminary Data Analysis

Table 1 shows the results of the descriptive analyses. All the measures had relatively adequate dispersions, and the accuracy rates ranged from 56.9% to 86.3%. The indices of skewness and kurtosis indicated the normality across different measures. The correlational matrix (Table 2) presents the results of bivariate correlations between prosodic sensitivity, oral vocabulary, and English word-level literacy. The findings demonstrated that all measurements were significantly correlated with each other. Notably, English oral vocabulary was correlated positively with stress, English word reading, and English word spelling (*r* ranges from .157 to .549, *ps* < .01).

### 3.2. Prosodic Transfer in English Word Reading and Word Spelling

**Figure 2:**
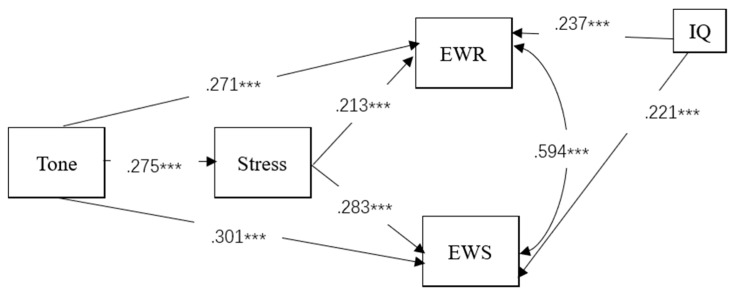
Standardized factor loadings on the path routes between tone and English word-level literacy (the best-fitting model). *** *p* < .001.

To explore whether tone sensitivity has direct and/or indirect effects on English word reading and spelling via lexical stress, we first tested the full model with IQ being controlled. The model was also evaluated with bootstrap confidence intervals using 5000 samples. Criteria for adequate fit are as follows: values greater than .95 on the comparative fit index (CFI) and greater than .90 on the goodness-of-fit index (GFI); and values below .085 on the root-mean-square error of approximation (RMSEA) and below .08 on the standardized root-mean-square residual (SRMR) (Hu and Bentler 1999). As shown in Table 3, χ2/df, SRMR, CFI, and TLI values met the cutoff criteria, but the RMSEA value slightly exceeded the reference value. The model was accepted considering that some authors suggested a less strict criterion for RMSEA value as a mediocre fit between .08 and .10 (Browne and Cudeck 1993). Standardized estimates of factor loadings of the full model are depicted in Figure 2. Inspection of the path weights indicated that all the routes were significant (*p* < .001).

Next, we tested the indirect tone transfer model A by fixing the direct pathway from lexical tone to English word reading to zero, and indirect model B by fixing the direct pathway from lexical tone to English word spelling to zero, respectively, and lastly, we tested the indirect model C by fixing the two direct pathways from tone to English word reading and spelling to zero. As summarized in Table 3, the latter three indirect models exhibited poor model fits. Hence, the full model was preferred as the best-fitting model. In the full model, tone directly predicted English word reading (*β* = .271, *p* < .001) and word spelling (*β* = .301, *p* < .001). There were also significant paths from tone awareness to stress sensitivity (*β* = .275, *p* < .001), and from stress sensitivity to English word reading (*β* = .213, *p* < .001) and spelling (*β* = .283, *p* < .001). These paths indicated that Chinese lexical awareness also indirectly affected English word reading and spelling through English stress.

### 3.3. The Role of Oral Vocabulary in the Prosodic Transfer Hypothesis

**Figure 3:**
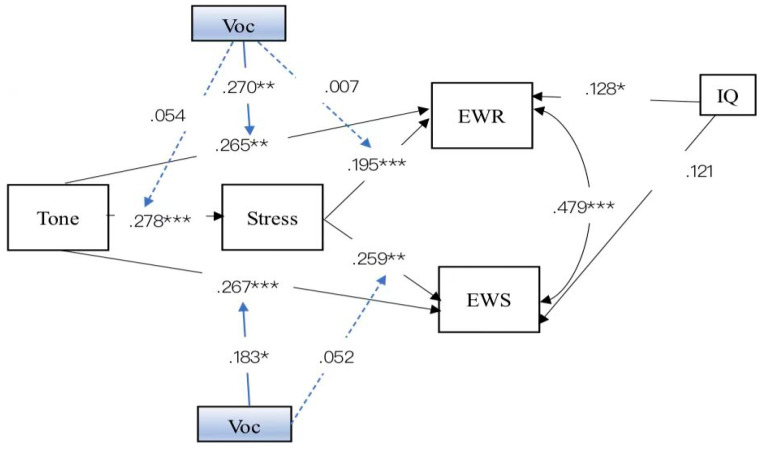
The moderated mediation model of tone, stress, vocabulary, and English word-level literacy with standardized factor loadings. Solid lines represent statistically significant relations whereas dashed lines represent nonsignificant relations. * *p* < .05, ** *p* < .01, *** *p* < .001.

**Figure 4:**
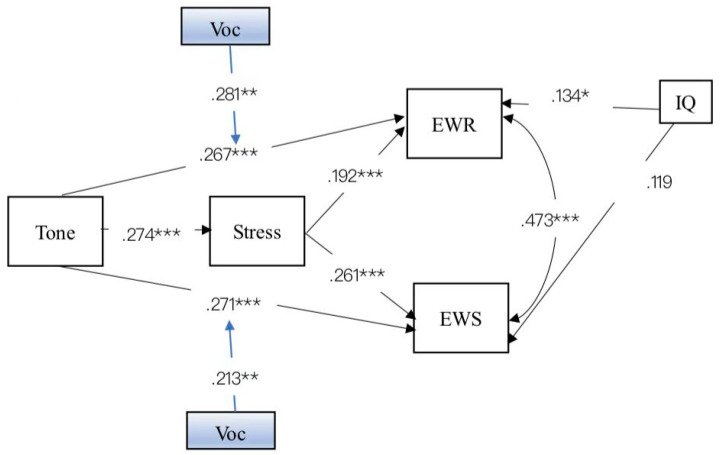
The respecified moderated mediation model of tone, stress, vocabulary, and English word-level literacy with standardized factor loadings. * *p* < .05, ** *p* < .01, *** *p* < .001.

To further assess the possible moderating effects of oral vocabulary, the latent moderated structural equation models (LMS) with bootstrap confidence intervals using 5000 samples were conducted to estimate the conditional effects of tone on performance through vocabulary at low (−1 *SD*), mean (0 *SD*), and high (+1 *SD*) levels of value. Lexical tone, stress, and oral vocabulary tasks were standardized prior to analysis. We included moderation from vocabulary along the pathways from tone to stress (vocabulary*tone→stress), to English word reading and English word spelling paths (vocabulary*tone→English word reading and spelling), and moderation from vocabulary along the pathway from stress to English word reading and word spelling (vocabulary*stress→English word reading and spelling) (shown in Figure 3). Based on cutoff criteria indicative of excellent and adequate fit, respectively: (a) comparative fit index (CFI) and Tucker-Lewis index (TLI) ≥ .95 and ≥ .90, respectively; (b) root-mean-square error of approximation (RMSEA) ≤ .085; and (c) standardized root-mean-square residual (SRMR) ≤ .08 (Hu and Bentler 1999), the indices of the model fit were good: χ2(2, *N* = 218) = 2.42, *p* = .298 (CFI = .998; TLI = .986; RMSEA = .03; SRMR = .021); χ2/df = 1.21 (shown in Table 4). The path coefficients of the structural model are shown in Table 5. Regarding the moderator of oral vocabulary, we found that it cannot predict stress (*β* = .054, *p* = .358), and that the interaction of stress and vocabulary did not predict English word reading (*β* = .007, *p* = .888) nor word spelling (*β* = .052, *p* = .317). Only the interaction of tone and oral vocabulary predicted English word reading (*β* = .270, *p* < .01) and spelling (*β* = .183, *p* < .05). It indicated that oral vocabulary only moderated the direct pathway from tone to English word-level literacy. Then, the insignificant pathways (from the interaction of tone and vocabulary to stress, from the interaction of stress and vocabulary to English word reading and spelling) were removed to further respecify the model (shown in Figure 4). As summarized in Table 4, the results of the model fit indices demonstrated that the respecified model fit was acceptable, χ2(4, *N* = 218) = 1.049, *p* = .370 (CFI = .999; TLI = .997; RMSEA = .015; SRMR = .030); χ2/df = 2.27. Then, a chi-square difference test between the two nested models indicated that the differences in chi-square values were not significant, Δχ2 (1, *N* = 218) = .727, *p* = .39 >.05. The fit of the respecified model was equal to the fit of the baseline model. Therefore, the respecified model, the more parsimonious model was favored as the best-fitting model (Schermelleh-Engel et al. 2003).

**Figure 5:**
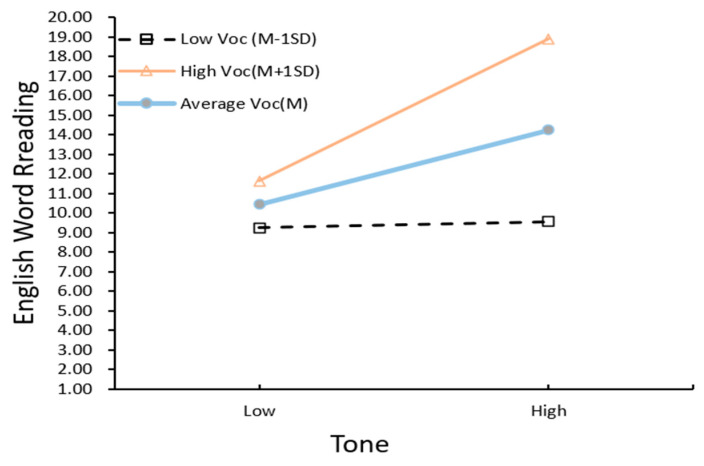
Performance of Grade 4 English word reading predicted by Chinese tone perception at low, average, and high levels of oral vocabulary scores. The x-axis depicts tone and the y-axis represents English word reading performance at low (−1 SD), medium (0 SD), and high (+1 SD) levels of oral vocabulary.

**Figure 6:**
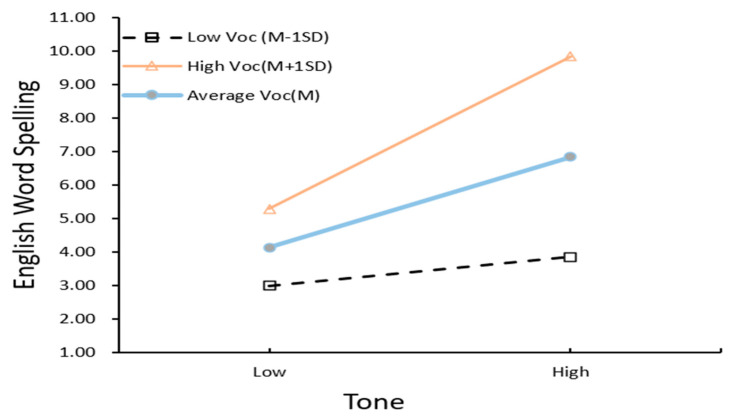
Performance of Grade 4 English word spelling predicted by Chinese tone perception at low, average, and high levels of oral vocabulary scores. The x-axis depicts tone and the y-axis represents English word reading performance at low (−1 SD), medium (0 SD), and high (+1 SD) levels of oral vocabulary.

Regression weights of the respecified moderated mediation model are shown in Table 6. The equation of the moderated direct pathway from tone to English word reading: $Y_{1}$ = (1.9 + 1.736Voc) Tone + 2.928Voc + 12.327, and from tone to English word spelling: $Y_{2}$ = (1.352 + .925Voc) Tone + 2.071Voc + 5.486. Tone perception by vocabulary interaction was further probed using simple slopes analysis (Aiken and West 1991). Figure 5 and Figure 6 plot the strengths of the tone–English word-level literacy relationship as a function of oral vocabulary.

To further verify the direct and indirect contributions of tone awareness to English word reading and spelling proficiency, standardized direct, indirect, and total effects of Chinese tone sensitivity were computed. Table 7 presents the direct, indirect, and total effects of Chinese tone perception on English word-level literacy varying with English oral vocabulary. Taken together with the simple slope analyses, the results indicated that the direct effect of tone on English word reading and word spelling was significant for children with vocabulary scores at mean (*β* = 1.9, *p* < .001 for English word reading; *β* = 1.352, *p* < .001 for English word spelling) and high levels (+1 *SD* above the mean) (*β* = 3.636, *p* < .001 for English word reading; *β* = 2.277, *p* < .001 for English word spelling). It also should be noted that the conditional positive direct effect of tone on L2 word reading was more robust than on L2 word spelling. In contrast, for children with oral vocabulary scores lower than 1*SD* below the mean, changes in levels of vocabulary did not exert significant influences on English word reading and spelling (*β* = .163, *p* = .756 > .05 for English word reading; *β* = .428, *p* = .286 > .05 for English word spelling). Notably, the differences in the direct effect of tone of English word-level literacy for vocabulary as a moderator at high and low values were pronounced (Δ*β* = 3.472, *p* < .001 for English word reading; Δ*β* = 1.849, *p* < .001 for English word spelling). Furthermore, the total effects of tone on English word reading were marginally significant (*β* = .537, *p* = .052) and those on word spelling were significant (*β* = .785, *p* < .01) for children with vocabulary scores lower than 1 *SD* below the mean.

To summarize, Chinese lexical tone had both direct and indirect effects on English word reading and spelling through lexical stress. English oral vocabulary significantly moderated the direct pathway from tone to English word-level literacy skills. This relationship became strengthened as oral vocabulary levels increased. Students with an average- or high-level of English oral vocabulary can benefit most from lexical tone awareness in propelling English word reading and spelling skills, while students with inadequate English oral vocabulary can hardly benefit from lexical tone awareness in shaping English word-level literacy.

## 4. Discussion

The current study highlighted the role of tone awareness and its direct and indirect cross-linguistic contribution to English word reading and word spelling through English stress among Chinese children at the transitional stage of reading. In addition, it emphasized the possible moderating role of oral vocabulary in the cross-linguistic relationship between L1 tone and L2 word-level literacy. Specifically, the strength of the direct linkage between L1 tone and L2 word-level literacy was proportional to vocabulary levels such that the cross-linguistic benefit from improved L1 tone awareness to L2 word reading and spelling is most salient among children with L2 oral vocabulary proficiency above average levels. In contrast, such benefits were shown to stagnate for children with low vocabulary proficiency. Taken together, our findings presented initial evidence for the direct and indirect cross-linguistic effects of tone in L2 word-level literacy skills through stress with the inclusion of word spelling performance as the outcome among Chinese–English bilingual learners and further endorsed the moderating effect of oral vocabulary.

### 4.1. Direct and Indirect Contributions of Tone Awareness to English Word Reading and Word Spelling

The first research question addressed the relationship between tone, stress, and English word-level literacy skills. The findings generated an interesting pattern of the prosodic transfer on English word reading and spelling outcomes. We noted that tone can both directly and indirectly contribute to English word reading and spelling through stress. This finding was consistent with the previous studies implicating a pivotal role of Cantonese tone sensitivity in facilitating English word reading and reading comprehension through stress (Choi et al. 2016, 2019b; Tong et al. 2016) and expanded the utility of prosodic transfer hypothesis among speakers of Mandarin with a less complex tonal system. The results provided further evidence for the role of prosody, particularly lexical stress, in explaining individual differences in L2 word reading. As noted in the literature review, prosody may facilitate word reading by providing children with cues to syntactic parsing to ‘chunk’ spoken language into comprehensible syntactic units (Gleitman et al. 1989; Kuhn and Stahl 2003). Such chunking can reduce memory load for efficient storage of information (Koriat et al. 2002; Speer et al. 1993). Sensitivity to lexical stress can also spare more cognitive resources from low-level processes such as segmentation to be focused on word reading (Choi et al. 2016; Tong et al. 2016). In addition, the prosodic transfer from tone to stress in L2 word-level literacy suggested a close association between L1 tone and L2 stress, which may arise from the shared acoustic feature (fundamental F0) and neural networks underlying speech perception (Gussenhoven 2004; Patel 2011, 2014), as well as the linguistic functions in distinguishing meanings (Cutler and Chen 1997, p. 165).

Critically, our study extended previous findings by showing that lexical prosody affects not only L2 word reading but also word spelling among Chinese–English bilinguals, which was consistent with previous studies on monolinguals (Gutiérrez-Palma et al. 2019). Spelling is a highly analytical process embracing phonological awareness, letter knowledge, and vocabulary knowledge (Shahar-Yames and Share 2008). In the spelling process, children first need to decode the sound into phonological constituents; utilize grapheme–phoneme correspondence rules to convert sound inputs into every corresponding letter, and at the same time attend to shapes and correct sequences (Shahar-Yames and Share 2008). Prosodic sensitivity may enhance word identification by locating word beginnings and guiding segmentation, which in turn help the recall of letter–sound knowledge in word spelling and aid the formation of the correct sequence and shape of the letter (Cutler and Norris 1988; Vroomen et al. 1998). In addition, prosodic sensitivity in Chinese and English can influence semantic information of the words (Deng et al. 2019; Tong et al. 2015). Since paired semantic information can additionally strengthen the phonological–orthographic associations and facilitate spelling accuracy (e.g., Hilte and Reitsma 2011; Ouellette 2010). In that sense, prosodic sensitivity may contribute to word spelling through facilitating word meaning construction.

Taken together, the significance of prosodic sensitivity in L2 word-level literacy suggests that suprasegmental phonological awareness should be taken into consideration in the theoretical account of early word-level literacy development across languages. The current study also corroborates the notion that phonological awareness seems to be a universal construct, regardless of typological distances (Shany et al. 2010). Chinese and English are two prosodically distinct language pairings, and the principal ways for prosodic cues to distinguish meaning are different (Cutler and Chen 1997, p. 165), however, these distinct linguistic features did not deter the prosodic transfer.

Intriguingly, the observed significant direct pathway from tone to L2 word reading in the current study was not shown to be evident in previous studies (e.g., Choi et al. 2016; Tong et al. 2016), which may attribute to the reading profiles of the participating learners. In Choi et al.’s (2016), and Tong et al. (2016)’s studies, the recruited participants were second graders. In contrast, the participants in the current study were fourth graders with higher language and literacy competencies. Such a discrepancy may induce the variations in the pathways driving the tone transfer.

### 4.2. The Moderator of Oral Vocabulary in the Cross-Linguistic Relation between Tone and L2 Word-Level Literacy

The unique contribution of the current study was the examination of how extant L2 oral language moderated the prosodic transfer in L2 word-level literacy among Chinese–English bilinguals. The findings showed that tone sensitivity could exert a more pronounced direct effect on L2 word reading and spelling of those learners with average and high vocabulary levels, and that such a relation was stronger for English word reading than word spelling. It implied that Chinese children with proficient English oral vocabulary could benefit more from L1 tone awareness in the formation of L2 word reading than L2 word spelling. In contrast, the conditional direct effect of tone on L2 word-level literacy was flat among those with low vocabulary scores. These findings extended previous cross-language transfer models (Chung et al. 2019; Cummins 1981, 2012; Koda 2008) by showing that cross-phonological awareness transfer involves units of analyses varying in complexity from phonological grain sizes beyond segments to lexical representations in the mental lexicon, and that it is determined jointly by multiple factors; that is, the interaction of oral vocabulary and tone perception influences the degree to which tone awareness impacts L2 word-level literacy. It also indicates the threshold for sufficient L2 oral vocabulary to allow for cross-linguistic prosodic transfer to occur. Specifically, the richness of a child’s lexical encoding of prosodic information across syllables may be uniquely important only among children at higher or average ranges of vocabulary skills. It is well recognized that increasing vocabulary size triggers the need to specify phonological representations into finer units to support reading (Metsala and Ehri 1998). To be specific, as vocabulary grows, children need to distinguish words previously stored in the mental lexicon from new words for successful recognition. In this process, children develop their ability to manipulate and perceive distinct phonological elements. The current finding extended lexical restructuring into the bilingual domain, and corroborated that such lexical restructuring processes may accelerate children’s detailed recognition mechanism for lexical representations to promote suprasegmental phonological awareness. Different from the lexical restructuring account which posited vocabulary as the precursor and protracted phonological restructuring dependent on vocabulary growth, our study, that investigated Chinese–English bilinguals, found a concurrent interaction between vocabulary and suprasegmental phonological awareness. This discrepancy may stem from the characteristics that prosodic sensitivity refers to the rhythmic patterning of speech and it may have cognitive and linguistic overlaps with oral vocabulary. Importantly, our study specified the exact role of oral vocabulary in moderating prosodic transfer in word-level literacy among Chinese–English bilingual children. Precisely, children with high oral vocabulary skills may have better-specified lexical representations to promote suprasegmental phonological awareness; thereby, they can benefit more from L2 suprasegmental phonological awareness in shaping L2 word reading and spelling skills. Notably, the conditional direct effects of tone on English word reading and word spelling skills were not significant among children with low oral proficiency. It implied that the bonus of lexical tone in L2 word spelling seems to be more parsimonious for Chinese–English bilingual children with under-developed oral vocabulary. Similar findings of the absence of the direct tone transfer in L2 literacy have been observed (Choi et al. 2016, 2019b; Tong et al. 2016) among second graders. Together with the current findings, the absence of the direct tone transfer may be partly due to the reading profiles of the second graders recruited in previous studies. Precisely, second graders may not reach the threshold for sufficient literacy experiences in oral vocabulary to enable L1 prosodic transfer in L2 reading development. It indicates that the strength of the prosodic transfer in L2 word-level literacy is impacted by oral vocabulary. The facilitative interaction between vocabulary and prosodic sensitivity needs to consider readers’ profile of oral vocabulary.

Intriguingly, we noted that L2 oral vocabulary failed to moderate the relation between tone and stress, and between stress and English word-level literacy. The pattern of findings needs further interpretation as well. As mentioned above, the similarities between lexical tone and stress lie in the fundamental perceptual mechanism of F0 tracking and linguistic functions for distinguishing meanings (Cutler and Chen 1997, p. 165; Gussenhoven 2004; Patel 2011, 2014). These similarities remain stable, and the strength bears no connections with oral vocabulary. Therefore, oral vocabulary may not moderate the cross-linguistic prosodic connections. In terms of the absence of the moderating effect of oral vocabulary on the pathway from stress to L2 word-literacy attainment, it indicates that within-language prosodic strength in reading is relatively stable compared with cross-linguistic prosodic strength, and is not proportional to vocabulary levels. It pinpointed the complexity of cross-language transfer models (Chung et al. 2019).

Collectively, the current study provided evidence that L1 prosodic skills (lexical tone awareness) contributed to L2 word-level literacy both directly and indirectly through L2 stress sensitivity. In particular, our study implicates a pivotal role of Chinese lexical tone awareness in L2 word spelling, which extends the utilities of the prosodic transfer hypothesis in the outcome of L2 word spelling. Additionally, our study revealed a spontaneous cross-linguistic tone transfer among children with high L2 oral vocabulary proficiency, while that cross-linguistic strength was not salient for children with low oral vocabulary skills. The pattern of findings underscored the complexity of the factors underlying cross-language transfer among emerging bilingual learners at the transitional stages from “learning to read” to “reading to learn” (Chall 1996). Importantly, the current study pointed to a precise threshold for sufficient L2 oral vocabulary proficiency to enable L1 tone transfer to occur in L2 word-level literacy attainment.

## 5. Implications

The study informed some pedagogical practices in Chinese–English bilingual learners at the transitional stages from “learning to read” and “reading to learn”. First, explicit prosodic awareness instruction may be needed to help children develop English word-level literacy prerequisites. Students can be instructed to make distinctions as to the different rhythmic patterning of the sound units. Tone awareness may not be directly transferred to L2 literacy performance among children without sufficient oral language proficiency. However, it can consolidate its link with L2 prosodic foundations for literacy attainment. Second, word reading and spelling instruction should be connected at a developmental sequence. The study endorsed the utilities of Chinese lexical tone perception and English stress sensitivity in both L2 word reading and word spelling. This predictive factor should be afforded greater importance in models of literacy development. Good performance of word reading could help learners to establish foundational links between sound, print, and meaning, through which children can integrate all of the components in the spelling task. Lin et al. (2017) ascertained the role of word spelling in enhancing the linkage between English word reading and the predictors of reading. In that sense, word spelling instruction should also be afforded much attention. Furthermore, educators must be aware of students’ heterogeneous linguistic profiles and ponder over the most effective instruction catering to learners’ oral language profiles. Children with adequate oral vocabulary skills can benefit more from L1 lexical tone awareness to support L2 literacy performance. Therefore, educational practitioners could design teaching activities specific to this group to foster children’s awareness of prosodic patterns at the early stage of second language learning. Conversely, those with low vocabulary skills cannot benefit directly from L1 prosodic awareness training. In that way, teachers may incorporate oral vocabulary skills (along with stress instruction) into early reading instruction to support developing L2 literacy skills.

## 6. Limitations and Guidelines for Future Research

Several limitations should be considered in the interpretation of the results obtained in this study. To begin with, the present study focused only on a particular developmental period: the middle of fourth grade. Further research using longitudinal design with autoregressive techniques and latent growth model analyses would allow us to test hypotheses about the potential cause–effect relationships between prosodic sensitivity, vocabulary, and children’s literacy skills, and about whether these patterns shift at different points in development. Furthermore, in testing the threshold for language proficiency to enable or debilitate the transfer of higher-level reading constructs, we also need to capture Chinese (L1) oral vocabulary. The current study only tapped into the moderating effect of L2 oral vocabulary. We argue that L1 oral vocabulary knowledge should also be underlined in the models of prosodic transfer in L2 reading development and offer a new theoretical model for future attempts at replication. Lastly, we only assessed prosodic transfer in the L1 (Chinese)-to-L2 (English) direction, which may constrain our ability to generalize the pattern of such transfer onto readers outside of China. The generalization of the current findings to readers of other orthographies needs to be conducted with caution. Studies to further extend our findings in readers outside of China are warranted.

## Figures and Tables

**Table 1:** Descriptive Statistics of all Measures.

Variable	Min	Max	M	SD	α	Skewness	Kurtosis
IQ (60)	10	53	36.84	8.11	.801	−.786	.722
Tone (10)	2	10	8.63	2.04	.721	−1.624	1.796
Stress (10)	1	10	5.69	2.01	.700	.085	−.630
Voc (20)	2	20	13.81	3.54	.788	−.489	−.138
EWR (36)	1	35	17.55	7.32	.755	.054	−.270
EWS (20)	.5	20	8.75	5.02	.863	.294	−.941

Note: N = 224; The maximum test scores are provided in parentheses. IQ = non-verbal ability; Tone = Chinese tone sensitivity; Stress = English lexical stress sensitivity; Voc = English oral vocabulary; EWR = English word reading; EWS = English word spelling.

**Table 2:** Bivariate Correlations between IQ, Tone, Stress, Oral vocabulary and Word-level Literacy.

	1	2	3	4	5	6
IQ	-					
Tone	.293 **	-				
Stress	.183 **	.268 **	-			
Voc	.420 **	.380 **	.157 *	-		
EWR	.345 **	.349 **	.302 **	.526 **	-	
EWS	.314 **	.411 **	.380 **	.549 **	.691 **	-

Note: IQ = non-verbal ability; Tone = Chinese tone sensitivity; Stress = English lexical stress sensitivity; Voc = English oral vocabulary; EWR = English word reading; EWS = English word spelling. * *p* < .05, ** *p* < .01.

**Table 3:** Goodness-of-fit indexes and comparisons of different models of the relation between Chinese lexical tone awareness and English word-level literacy among Grade 4 Chinese–English bilingual children.

Model	χ2	df	CFI	TLI	RMSEA	SRMR	AIC	BIC
Full Model	2.91	1	.991	.922	.092	.031	3348.456	3396.344
Indirect Model A	18.45	2	.926	.665	.191	.081	3362.015	3406.482
Indirect model B	21.77	2	.911	.598	.209	.089	3365.313	3409.780
Indirect model C	25.03	3	.900	.701	.180	.100	3366.574	3407.621

**Table 4:** Goodness-of-fit Indexes and Comparisons of Different Models of the Relation between Chinese Lexical Tone Awareness and English Word-level Literacy among Grade 4 Chinese Learners of English.

Model	χ2	df	CFI	TLI	RMSEA	SRMR	AIC	BIC	Δχ2	Δdf
Original Model	2.42	2	.998	.986	.003	.021	2928.227	3002.686	-	-
Respecified Model	3.147	3	.999	.997	.015	.030	296.071	3021.480	.727	1

**Table 5:** Moderated Structural Equation Modeling: Effects of Tone, Stress, Vocabulary, Tone*Stress Interaction, Stress*Vocabulary Interaction on English Word Reading and Spelling Performance.

Paths	β	βˆ	SE	C.R.(z)	*p*
Stress←Tone	.278	.278	.072	3.852	.000
Stress←Voc	.064	.064	.070	.919	.358
Stress←Tone*Voc	.047	.054	.072	.743	.457
EWR←Tone	1.906	.265	.067	3.975	.000
EWR←Voc	2.898	.402	.060	6.681	.000
EWR←Tone*Voc	1.694	.270	.095	2.837	.005
EWR←Stress	1.401	.195	.057	3.407	.001
EWR←Stress*Voc	.057	.007	.053	.141	.888
EWS←Tone	1.346	.267	.064	4.171	.000
EWS←Voc	2.079	.412	.062	6.622	.000
EWS←Tone*Voc	.803	.183	.091	2.004	.045
EWS←Stress	1.311	.259	.056	4.649	.000
EWS←Stress*Voc	.280	.052	.052	1.001	.317

**Table 6:** Moderated Structural Equation Modeling: Effects of Tone, Stress, Vocabulary, Tone*Stress Interaction on English Word Reading and Spelling Performance.

Paths	β	βˆ	SE	C.R.(z)	*p*
Stress←Tone	.273	.274	.054	5.077	.000
EWR←Tone	1.900	.267	.066	4.078	.000
EWR←Voc	2.928	.412	.061	6.766	.000
EWR←Tone*Voc	1.736	.281	.092	3.057	.002
EWR←Stress	1.366	.192	.057	3.395	.001
EWS←Tone	1.352	.271	.061	4.414	.000
EWS←Voc	2.071	.414	.061	6.741	.000
EWS←Tone*Voc	.925	.213	.077	2.767	.006
EWS←Stress	1.308	.261	.056	4.648	.000

**Table 7:** Conditional Effects of Tone on English Word Reading and Word Spelling.

		EWR			EWS		
	Perceived Value of Vocabulary	Direct [95%CI]	Indirect [95%CI]	Total [95%CI]	Direct [95%CI]	Indirect [95%CI]	Total [95%CI]
Tone	(low) −1*SD*	.163 [−1.108, 1.257]	.373 ** [.164, .701]	.537 [−.675, 1.621]	.428 [−.155, 1.020]	.358 ** [.174, .618]	.785 ** [.172, 1.359]
	(average) 0*SD*	1.9 *** [1.038, 2.956]		2.273 *** [1.449, 3.306]	1.352 *** [.805, 2.012]		1.71 *** [1.155, 2.373]
	(high) 1*SD*	3.636 *** [2.180, 5.603]		4.009 *** [2.573, 5.931]	2.277 *** [1.262, 3.491]		2.634 *** [1.637, 3.831]
Stress		-	1.366 ** [.630, 2.132]	1.366 ** [.630, 2.132]	-	1.308 *** [.760, 1.842]	1.308 *** [.760, 1.842]

** *p* < .01, *** *p* < .001.

## Data Availability

The data presented in this study are available on request from the corresponding author.

## References

[B1-jintelligence-10-00114] Aiken Leona S., West Stephen G. (1991). Multiple Regression: Testing and Interpreting Interactions.

[B2-jintelligence-10-00114] Atwill Kim, Blanchard Jay, Christie James, Gorin Joana S., García Hermán (2010). English-language learners: Implications of limited vocabulary for cross-language transfer of phonemic awareness with kindergartners. Journal of Hispanic Higher Education.

[B3-jintelligence-10-00114] Browne Michael W., Cudeck Robert, Bollen Kenneth A., Long J. Scott (1993). Alternative ways of assessing model fit. Testing Structural Equation Models.

[B4-jintelligence-10-00114] Burnham Denis, Kim Jeesun, Davis Chris, Ciocca Valter, Schoknecht Colin, Kasisopa Benjawan, Luksaneeyanawin Sudaporn (2011). Are Tones Phones?. Journal of Experimental Child Psychology.

[B5-jintelligence-10-00114] Bus Adriana G., van IJzendoorn Marinus H. (1999). Phonological Awareness and Early Reading: A Meta-Analysis of Experimental Training Studies. Journal of Educational Psychology.

[B6-jintelligence-10-00114] Chall Jeanne (1983). Stages of Reading Development.

[B7-jintelligence-10-00114] Chall Jeanne (1996). Stages of Reading Development.

[B8-jintelligence-10-00114] Choi William, Tong Shelly Xiuli, Cain Kate (2016). Lexical prosody beyond L1 boundary: Chinese lexical tone sensitivity predicts English reading comprehension. Journal of Experimental Child Psychology.

[B9-jintelligence-10-00114] Choi William, Tong Shelly Xiuli, Singh Leher (2017). From lexical tone to lexical stress: A cross-language mediation model for Cantonese children learning English as a second language. Frontiers in Psychology.

[B10-jintelligence-10-00114] Choi William, Tong Shelly Xiuli, Samuel Arthur G. (2019a). Better than native: Tone language experience enhances English lexical stress discrimination in Cantonese-English bilingual listeners. Cognition.

[B11-jintelligence-10-00114] Choi William, Tong Shelly Xiuli, Deacon Helene (2019b). From Cantonese lexical tone awareness to second language English vocabulary: Cross-language mediation by segmental phonological awareness. Journal of Speech Language and Hearing Research.

[B12-jintelligence-10-00114] Chung Sheila Cira, Chen Xi, Geva Esther (2019). Deconstructing and Reconstructing Cross-Language Transfer in Bilingual Reading Development: An Interactive Framework. Journal of Neurolinguistics.

[B13-jintelligence-10-00114] Cummins Jim, California State Department of Education (1981). The role of primary language development in promoting educational success for language minority students. Schooling and Language Minority Students: A Theoretical Framework.

[B14-jintelligence-10-00114] Cummins Jim (2012). The intersection of cognitive and sociocultural factors in the development of reading comprehension among immigrant students. Reading and Writing.

[B15-jintelligence-10-00114] Cutler Anne, Chen Hsuan-Chin (1997). Lexical tone in Cantonese spoken-word processing. Perception and Psychophysics.

[B16-jintelligence-10-00114] Cutler Anne, Norris Dennis (1988). The role of strong syllables in segmentation for lexical access. Journal of Experimental Psychology.

[B17-jintelligence-10-00114] Deng Qinli, Choi William, Tong Xiuli (2019). Bidirectional cross-linguistic association of phonological skills and reading comprehension: Evidence from Hongkong Chinese–English bilingual readers. Journal of Learning Disabilities.

[B18-jintelligence-10-00114] Dunn M. Lloyd, Dunn Leota M. (1997). PPVT-III: Peabody Picture Vocabulary Test.

[B19-jintelligence-10-00114] Farnia Fataneh, Geva Esther (2011). Cognitive correlates of vocabulary growth in English language learners. Applied Psycholinguistics.

[B20-jintelligence-10-00114] Fitzgerald Jill, Shanahan Timothy (2000). Reading and writing relations and their development. Educational Psychologist.

[B21-jintelligence-10-00114] Fry Dennis Butler (1958). Experiments in the perception of stress. Language and Speech.

[B22-jintelligence-10-00114] Gleitman Lila, Gleitman Henry, Landau Barbara, Wanner Eric, Galaburda Albert M. (1989). Great expectations. From Reading to Neurons.

[B23-jintelligence-10-00114] Gottardo Alexandra, Yan Bernice, Siegel Linda S., Wade-Woolley Lesly (2001). Factors related to English reading performance in children with Chinese as a first language: More evidence of cross-language transfer of phonological processing. Journal of Educational Psychology.

[B24-jintelligence-10-00114] Gottardo Alexandra, Collins Penelope, Yan Bernice, Siegel Linda S., Gu Yan (2006). Relationships between first and second language phonological processing skills and reading in Chinese–English speakers living in English-speaking contexts. Educational Psychology.

[B25-jintelligence-10-00114] Gussenhoven Carlos (2004). The Phonology of Tone and Intonation.

[B26-jintelligence-10-00114] Gutiérrez-Palma Nicolás, Naranjo Nieves Valencia, Justicia-Galiano María José, Fernández Carpio, Villa María de la (2019). Beyond phonological awareness: Stress awareness and learning word spelling. Learning and Individual Differences.

[B27-jintelligence-10-00114] Hilte Maartje, Reitsma Pieter (2011). Activating the Meaning of a Word Facilitates the Integration of Orthography: Evidence from Spelling Exercises in Beginning Spellers. Journal of Research in Reading.

[B28-jintelligence-10-00114] Holliman Andrew J., Palma Nicolás Gutiérrez, Critten Sarah, Wood Clare, Cunnane Helen, Pillinger Claire (2017). Examining the Independent Contribution of Prosodic Sensitivity to Word Reading and Spelling in Early Readers. Reading and Writing.

[B29-jintelligence-10-00114] Hu Li-tze, Bentler Peter M. (1999). Cutoff criteria for fit indexes in covariance structure analysis: Conventional criteria versus new alternatives. Structural Equation Modeling: A Multidisciplinary Journal.

[B30-jintelligence-10-00114] Joshi Malatesha R. (2005). Vocabulary: A critical component of comprehension. Reading and Writing Quarterly: Overcoming Learning Difficulties.

[B31-jintelligence-10-00114] Kim Young-Suk Grace, Boyle Helen N., Zuilkowski Stephanie Simmons, Nakamura Pooja (2016). The Landscape Report on Early Grade Literacy Skills.

[B32-jintelligence-10-00114] Koda Keiko, Koda Keiko, Zehler Annette M. (2008). Impacts of prior literacy experience on second-language learning to read. Learning to Read Across Languages: Crosslinguistic Relationships in First- and Second-Language Literacy Development.

[B33-jintelligence-10-00114] Koda Keiko, Lü Chan, Zhang Dongbo, Chen Xi, Wang Qiuying, Luo Yang Cathy (2014). L1-Induced Facilitation in Biliteracy Development in Chinese and English. Reading Development and Difficulties in Monolingual and Bilingual Chinese Children.

[B34-jintelligence-10-00114] Koriat Asher, Greenberg Seth N., Kreiner Hamutal (2002). The extraction of structure during reading: Evidence from reading prosody. Memory and Cognition.

[B35-jintelligence-10-00114] Kuhn Melanie R., Stahl Steven A. (2003). Fluency: A Review of Developmental and Remedial Practices. Journal of Educational Psychology.

[B36-jintelligence-10-00114] Lee Kathleen, Chen Xi (2019). An emergent interaction between reading fluency and vocabulary in the prediction of reading comprehension among French immersion elementary students. Reading and Writing.

[B37-jintelligence-10-00114] Lervåg Arne, Aukrust Vibeke Grøver (2010). Vocabulary Knowledge is a Critical Determinant of the Difference in Reading Comprehension Growth between First and Second Language Learners. Journal of Child Psychology and Psychiatry.

[B38-jintelligence-10-00114] Lin Dan, Liu Yingyi, Sun Huilin, Wong Richard Kwok Shing, Yeung Susanna Siu-sze (2017). The pathway to English word reading in Chinese ESL children: The role of spelling. Reading and Writing.

[B39-jintelligence-10-00114] Metsala Jamie L., Ehri Linnea C. (1998). Spoken vocabulary growth and the segmental restructuring of lexical representations: Precursors to phonemic awareness and early reading ability. Word Recognition in Beginning Literacy.

[B40-jintelligence-10-00114] Ministry of Education of the People’s Republic of China (2021). Curriculum Criteria for Compulsory Education of English.

[B41-jintelligence-10-00114] Ouellette Gene (2010). Orthographic Learning in Learning to Spell: The Roles of Semantics and Type of Practice. Journal of Experimental Child Psychology.

[B42-jintelligence-10-00114] Patel Aniruddh D. (2011). Why would Musical Training Benefit the Neural Encoding of Speech? The OPERA Hypothesis. Frontiers in Psychology.

[B43-jintelligence-10-00114] Patel Aniruddh D. (2014). Can Nonlinguistic Musical Training Change the Way the Brain Processes Speech? The Expanded OPERA Hypothesis. Hearing Research.

[B44-jintelligence-10-00114] Protopapas Athanassios, Gerakaki Svetlana, Alexandri Stella (2006). Lexical and Default Stress Assignment in Reading Greek. Journal of Research in Reading.

[B45-jintelligence-10-00114] Riedel W. Brant (2007). The relation between DIBELS, reading comprehension, and vocabulary in urban first-grade students. Reading Research Quarterly.

[B46-jintelligence-10-00114] Schermelleh-Engel Karin, Moosbrugger Helfried, Muller Hans (2003). Evaluating the fit of structural equation models: Tests of significance and descriptive goodness-of-fit measures. Methods of Psychological Research.

[B47-jintelligence-10-00114] Schwanenflugel J. Paula, Benjamin Rebekah (2017). Lexical prosody as an aspect of oral reading fluency. Reading and Writing.

[B48-jintelligence-10-00114] Shahar-Yames Daphna, Share David L. (2008). Spelling as a Self-Teaching Mechanism in Orthographic Learning. Journal of Research in Reading.

[B49-jintelligence-10-00114] Shany Michael, Esther Geva, Liat Melech-Feder (2010). Emergent literacy in children of immigrants coming from a primarily oral literacy culture. Written Language and Literacy.

[B50-jintelligence-10-00114] Speer Shari R., Crowder Robert G., Thomas Lisa M. (1993). Prosodic structure and sentence recognition. Journal of Memory and Language.

[B51-jintelligence-10-00114] Stanovich Keith E., Siegel Linda S. (1994). Phenotypic performance profile of children with reading disabilities: A regression-based test of the phonological-core variable-difference model. Journal of Educational Psychology.

[B52-jintelligence-10-00114] Tong Xiuli, Tong Xiuhong, McBride-Chang Catherine (2015). Tune in to the tone: Lexical tone identification is associated with vocabulary and word recognition abilities in young Chinese children. Language and Speech.

[B53-jintelligence-10-00114] Tong Xiuli, He Xinjie, Deacon S. Hélène (2016). Tone matters for Cantonese-English bilingual children’s English word reading development: A unified model of phonological transfer. Memory and Cognition.

[B54-jintelligence-10-00114] Uwezo (2011). Are Our Children Learning?.

[B55-jintelligence-10-00114] Vroomen Jean, Tuomainen Jyrki, de Gelder Beatrice (1998). The Roles of Word Stress and Vowel Harmony in Speech Segmentation. Journal of Memory and Language.

[B56-jintelligence-10-00114] Wang Min, Yang Chen, Cheng Chenxi (2009). The contributions of phonology orthography and morphology in Chinese–English biliteracy acquisition. Applied Psycholinguistics.

[B57-jintelligence-10-00114] Wood Clare, Wade-Woolley Lesly, Holliman Andrew J., Wood Clare, Connelly Vincent (2009). Phonological awareness: Beyond phonemes. Contemporary Perspectives on Reading and Spelling.

[B58-jintelligence-10-00114] Woodcock W. Richard (1998). Woodcock Reading Mastery Test-Revised.

[B59-jintelligence-10-00114] Zhang Haomin (2016). Early language input and later reading development in Chinese as heritage language (CHL) learners. International Journal of Applied Linguistics.

[B60-jintelligence-10-00114] Zhang Haomin, Koda Keiko (2021). Early oral language in Chinese heritage language reading development. Foreign Language Annals.

[B61-jintelligence-10-00114] Zhang Houcan, Wang Xiaoping (1985). Raven’s IQ Reasoning Standardized Test.

[B62-jintelligence-10-00114] Zhang Haomin, Lin Jiexin, Cheng Xi, Wang Chichi, Wei Xiaobao (2022). Concurrent and longitudinal contributions of phonological awareness to early adolescent Chinese reading acquisition. The Journal of General Psychology.

